# Rectal Discomfort

**Published:** 1907-07-20

**Authors:** J. B. Dawson

**Affiliations:** House Surgeon to St. Mark's Hospital


### RECTAL DISCOMFORT.

By J. B. DAWSON, M.B., B.S.(Lond.), M.R.C.S., L.R.C.P., House Surgeon to St. Mark's Hospital.

From disinclination, partly of the patient and partly of the practitioner, conditions of rectal uneasiness more often than not are treated without proper or adequate examination. In private practice as much tact and care of the patient's feelings are required whilst making examination per rectum as per vaginam. It is a peculiar fact that many rectal conditions indicate their presence by various but persistent symptoms of a subacute nature rather than by those causing acute distress. In such cases the patient may allow of inspection of the anal region, but will probably demur to digital examination or a suggested use of the proctoscope. Nevertheless, unless full investigation be made, all treatment must necessarily be guesswork, and many conditions of the utmost gravity will escape diagnosis.

The expression "rectal discomfort" is used advisedly, for it indicates the commonest complaint of those suffering from pathological conditions of that viscus. Whilst the rectal troubles that commonly cause acute pain can be numbered on the fingers, those that give rise to persistent uneasiness are much more numerous, and lead to a train of mental symptoms or to sequelæ of a more local character, that render the patient a pitiable invalid.

### A Classification.

The following is an attempt at classification:—

1. *Parasites of the Rectum and Anal Region.*—The ascaris lumbricoides, though an inhabitant of the small gut, finds its way through the colon, and may be a cause of irritability and tenesmus.

The oxyuris vermicularis is a common cause of distress in children, leading them to irritate and finger the anus, and produces besides a series of mental and nutritional disorders.

Pediculi, especially in hirsute and dirty men, may produce intense itching; persistent scratching may produce excoriations, and initiate a pruritus of intractable character.

2. *Constipation*, due to hygienic carelessness, sedentary habits, or improper diet, is a cause of tenesmus and sense of weight in the perineum, symptoms often accompanied by abdominal pain. These are partly due to the dragging of a loaded iliac and pelvic colon, and possibly to an inflamed mucous membrane; the latter by an abundant excretion of mucus may produce a spurious diarrhœa.

3. *Hæmorrhoids.*—This is a condition so well known and so easy to observe by a proper examination that it needs small notice. Unless inflamed, thrombosed or prolapsed, piles rarely cause acute pain. Small piles which have to be sought for may, however, be the determining factor of a case of rectal uneasiness.

4. *Verrucæ and Tags.*—Warts and cutaneous tags around the anus may produce itching, and are often the cause of intense pruritus. Of themselves they are harmless, but by rendering it difficult for the patient to properly cleanse himself after defæcation they produce irritation. It should be borne in mind that a wart is a papilloma, and that papillomata, especially when exposed to friction, occasionally become malignant.

At this point it seems appropriate to point out the unsatisfactory and unclean custom in this country of using dry paper for the anal toilette. It is obviously impossible, even with good soft sanitary paper, to render the circumanal region clean after defæcation. All that is done is to smear fæces on the skin and then to assist their entry of the hair follicles by friction. Many so-called savage tribes set us an example in this matter by their habit of squatting in water and assisting the removal of fæces with grass or what not.

5. *Fistula in Ano.*—Again a common and well-recognised condition. Nevertheless a blind internal fistula either of the submucous or interstitial variety cannot be recognised without thorough and complete investigation.

### Ulcerations.

6. *Fissure and Ulcer.*—Conditions which often occasion severe pain, but which may indicate their presence by nothing more than discomfort. A little known fact is that there can be found running up from the crypts of Morgagni submucous, test-tube like pouches varying in length from $\frac{1}{8}$ to $\frac{1}{2}$ of an inch.* Around the mouth of these inflammation and ulceration is common. This of itself may cause distress, whilst the discharge originating there may be the unsuspected cause of pruritus and fissure. Indeed fissures, when traced upwards, may be found to lose themselves in an ulcerated patch. These submucous pouches may be the cause of rectal pain, apart from any destruction of the mucous surface of the bowel. It is not difficult to understand how they may act as a resthouse and incubation centre for the many pyogenic organisms swarming in the bowel. Rokitansky has drawn an interesting comparison between the varicose ulcer of the leg and the ulcer found in the rectum, accompanying piles. In both there is venous engorgement with corresponding diminution of resistance to invasion by bacteria, which the delicate mucous membrane can hardly be expected to withstand when the more resistant skin of the leg succumbs.

7. *Ulceration.*—By this is meant more extensive destruction of mucous membrane over a greater area of bowel. It may be simple, syphilitic, tubercular, or dysenteric in character, to discuss which at any length is outside our province. It may be of interest to say that the records of St. Mark's Hospital show that syphilitic ulceration, that is, a lesion due to destruction of tertiary specific deposits or to secondary ulceration is extremely rare.

Extensive ulceration of the bowel is usually the cause of intense discomfort and misery, and a condition which compels attention. Tubercular ulceration of the rectum proper produces symptoms of a more chronic and subacute kind. It is often associated with tubercular fistula and with phthisis.

8. *Stricture.*—The causes and symptoms cannot be here discussed, but it is of importance to note that constipation associated with bearing down pain referred to the lumbar region, and a sense of dragging during the act of defæcation may indicate the onset of contraction which is to end in stricture. It should be remembered that various gynæcological conditions may produce similar symptoms.

### New Growths.

9. *Neoplasms.*—(*a*) The rectal polypus is a common cause of tenesmus, straining at stool, frequent desire for defæcation, and a sense of incompletion of the act. In children straining at stool with the passage of blood and mucous is strongly indicative of the presence of a polypus. (*b*) *Carcinoma.*—Malignant growth of the rectum when it is of the stenosing variety, or when it has come to implicate surrounding pelvic structures, may cause intense pain; but a cancer may exist for many months and give no sensory symptoms other than those of rectal fullness, frequent desire to open the bowels, and sometimes aching pain when fæces are passed. It is a frequent occurrence to receive patients who have had attendant piles or fistula treated, but whose medical attendant has failed to recognise a more serious condition underlying. Probably if early recognition were commoner, many growths of the rectum would receive operative treatment while still innocent, or on the borderline between innocence and malignancy.

It is evident, therefore, that a variety of pathological phenomena may underlie symptoms which are not intense enough to obtrude themselves upon the patient, and compel his consultation of a medical man. It is by so much the more important that the public shall have no valid ground of complaint against the profession on the score of misdiagnosis of these diseases. Errors and omissions of diagnosis are almost always founded on insufficient examination, and it is very unwise to treat rectal disease or symptoms in any patient without making every effort to induce him to submit to local investigation.

* Wallis: "Diseases of the Rectum."

